# Multiphoton Intravital Microscopy of Mandibular Draining Lymph Nodes: A Mouse Model to Study Corneal Immune Responses

**DOI:** 10.3389/fimmu.2020.00039

**Published:** 2020-02-21

**Authors:** Maria J. Lopez, Yashar Seyed-Razavi, Takefumi Yamaguchi, Gustavo Ortiz, Victor G. Sendra, Deshea L. Harris, Arsia Jamali, Pedram Hamrah

**Affiliations:** ^1^Department of Ophthalmology, Center for Translational Ocular Immunology, Tufts Medical Center, Tufts University School of Medicine, Boston, MA, United States; ^2^Department of Ophthalmology, Harvard Medical School, Schepens Eye Research Institute/Massachusetts Eye and Ear Infirmary, Boston, MA, United States; ^3^Program in Immunology, School of Graduate Biomedical Sciences, Tufts University, Boston, MA, United States; ^4^Cornea Service, Department of Ophthalmology, Tufts New England Eye Center, Tufts Medical Center, Tufts University School of Medicine, Boston, MA, United States; ^5^Cornea Service, Department of Ophthalmology, Massachusetts Eye & Ear Infirmary, Harvard Medical School, Boston, MA, United States

**Keywords:** intravital imaging, multiphoton microscopy, corneal transplantation, mandibular draining lymph nodes, antigen presenting cells, kinetics, dendritic cells

## Abstract

Multiphoton intravital microscopy (MP-IVM) is a powerful tool to image cells *in vivo*. Its application in immunology research has opened new horizons, allowing intravital imaging of leukocytes at the single-cell level. A transparent cornea is vital to retain vision. As an immune privileged site, a rapid innate response to foreign antigens is crucial in clearing opportunistic bacterial and viral pathogens, and minimizing collateral structural damage to the cornea. Furthermore, dissecting the mechanisms and preventing the immunological rejection process after corneal transplantation is imperative to retain sight. Therefore, understanding the underlying mechanisms behind corneal immunity, specifically the process of antigen presentation and adaptive immunity in the mandibular draining lymph nodes (dLNs) *in vivo*, is crucial. Attempts of intravital imaging of mandibular dLNs have yielded little success to date, due to breathing artifacts and the location that is difficult to access. Herein, we present the first MP-IVM mouse model of the mandibular dLNs, utilizing transgenic mice in which CD11c^+^ cells are fluorescently labeled. Furthermore, we demonstrate that CD11c-YFP^+^ cells are localized mainly in the parafollicular cortex (T cell zone) and subcapsular area and are sparsely distributed in the follicular region (B cell zone) of mandibular dLNs during steady state. A significant increase in host CD11c-YFP^+^ cell density is noted at 14 and 21 days following allogeneic corneal transplantation, compared to steady state (*p* < 0.05). Moreover, allogeneic corneal transplantation results in increased host-derived CD11c-YFP^+^ cell mean speed and displacement in mandibular dLNs, compared to steady state (*p* < 0.001). The meandering index, an index for directionality, is significantly increased after allogeneic corneal transplantation at both 14 and 21 days, compared to steady state (*p* < 0.001). Taken together, our study demonstrates the necessary methodology required for intravital multiphoton imaging of the mandibular dLNs, allowing visualization of spatiotemporal kinetics of immune cells *in vivo*, and provides a window into the corneal immune reflex arc. This technique will be a powerful tool to investigate the pathogenesis of ocular immune and inflammatory diseases.

## Introduction

As a window to the foreign world, the immune privileged status of the cornea is crucial in maintaining transparency and vision in the face of constant exposure to the external environment (1–4). Following damage to the epithelial layer, a rapid, specific, and selective immune response is mounted against foreign antigens, including opportunistic bacterial and viral pathogens, as chronic keratitis can lead to structural alterations of the corneal stroma that may result in opacity, corneal scarring, and ultimately vision impairment (5).

Before the discovery of resident bone marrow-derived leukocytes, the cornea was long believed to be a collagenous tissue devoid of leukocytes. However, it is now acknowledged that the steady state mammalian cornea contains a heterogeneous populations of resident corneal leukocytes, including professional antigen-presenting cells (APCs), such as conventional dendritic cells (cDCs) and macrophages (6–8). cDCs are unique in their nature to prime T cells and induce antigen-specific immune responses or immunological tolerance in draining lymph nodes (dLNs) (9–11). Interestingly, in the ocular tissues, evidence suggests that antigens can also directly drain into the mandibular dLNs (12–14). Therefore, the investigation of the process of antigen presentation by corneal cDCs in the mandibular dLNs will provide understanding of underlying mechanisms behind corneal immunity, specifically the process of antigen presentation and adaptive immunity. Thus, we sought to study APCs in the mandibular dLNs *in vivo*, where the physiological conditions such as tissue oxygenation and temperature, and therefore tissue environment, are preserved.

Multiphoton intravital microscopy (MP-IVM) has significant advantages over other types of microscopy. It allows long-term imaging of biological processes, with minimal toxicity and photobleaching (15–17), and achieves high-contrast images at the cellular level in thick, non-transparent specimens with less potential photo-damage (18, 19). Furthermore, a distinct advantage of MP-IVM is that it provides high spatiotemporal resolution, allowing for cellular analysis of leukocytes, such as cDCs (20, 21), T cells, and B cells in their native anatomical context (22–24). Furthermore, interaction between different leukocytes during steady state and in pathological conditions have been described with MP-IVM (25–28).

MP-IVM has been widely used to image inguinal and popliteal LNs for lymphocyte trafficking in *in vivo* and *ex vivo* settings (20, 22, 23, 25, 29–31). However, to date, studies of the mandibular dLNs have only been attempted in *ex vivo* and *in vitro* studies (12, 27, 32, 33). Possible reasons why MP-IVM of the mandibular dLNs have as of yet been largely missing may be the difficultly in exposing the tissue and inability to properly stabilize it, leading to artifacts arising from breathing and pulsations from the beating heart. In the current study, we present for the first time, to our knowledge, the necessary steps to provide stable long-term MP-IVM imaging of the mandibular dLNs and reveal CD11c-YFP^+^ cell kinetics during steady state and following allogeneic corneal transplantation.

## Methods

### Animals

Six- to 8-week old male transgenic mice expressing yellow fluorescent protein (YFP) under the control of the CD11c promoter (C57BL/6 background; a kind gift of Dr. Michel C. Nussenzweig from Rockefeller University; called CD11c-YFP mice) (20) and transgenic T-Red mice selectively expressing DsRed in T cells (C57BL/6 background; a kind gift of Dr. Ulrich H. von Andrian, Harvard Medical School) (34) were bred in house. CD11c-YFP mice were used as recipients in our murine model of corneal transplantation. Age- and sex-matched wild-type (WT) BALB/c mice (Charles River Laboratory, Wilmington, MA, United States) served as corneal donors. The Schepens Eye Research Institute and Tufts Medical Center Animal Care and Use Committees approved the protocol. We treated all animals according to the ARVO Statement for the Use of Animals in Ophthalmic and Vision Research.

### Corneal Transplantation

To study the behavior of immune cells in diseased states of the cornea, we used a murine model of corneal allotransplantation (allogeneic) as previously described (35). Briefly, BALB/c mice were used as corneal donors; a 2.0-mm trephine was used to delimitate the donor button, which was excised with Vannas scissors (2 mm cutting edge; Fine Science Tools, Foster City, CA, United States) and transplanted into anesthetized CD11c-YFP transgenic mice. The host bed was prepared by excising 1.5 mm of the central cornea, previously demarcated using a 1.5 mm trephine. The donor cornea was secured to the host bed with eight interrupted 11-0 Nylon sutures (A9016N Surgical Specialties, Wyomissing, PA, United States). At the end of the procedure, antibiotic ointment (Erythromycin Ophthalmic Ointment USP, 0.5%, Bausch & Lomb Inc., Tampa, FL, United States) was applied to the cornea to reduce the risk of infections, and the eyelids were closed by performing a tarsorrhaphy for 3–5 days with 8-0 Nylon sutures (AA-0122 Surgical Specialties). The corneal sutures were removed 7 days after transplantation, and animals underwent MP-IVM after 14 and 21 days postoperatively.

### Multiphoton Intravital Microscopy

We used a custom-made multiphoton intravital microscope Ultima Pro Multiphoton Microscopy System, Bruker Technology, WI, United States) equipped with two Mai Tai Titanium-Sapphire lasers (Newport Spectra-Physics, Irvine, United States) for simultaneous coaxial illumination. One of the lasers was set at 850 nm wavelength (to visualize YFP^+^ cells) or 760 nm wavelength (to visualize DsRed^+^ cells), and the other laser was set at 900 nm wavelength for second harmonic generation to visualize the dLN capsule in a completely separated channel, a convenient tool to analyze the anatomical distribution of the cells within the dLN. Image acquisition was performed using Prairie View Software (Bruker Technology). A 20× water immersion objective (NA: 0.95, Olympus, XLUMPlanFL N, Tokyo, Japan) was used to capture images of the mandibular dLNs with volumes of 70 × 596 × 596 μm at 3 μm Z-steps with 30 s intervals over 30 min.

### Immunohistochemistry and Confocal Microscopy

Mandibular dLNs of CD11c-YFP mice were excised, fixed in 4% paraformaldehyde at room temperature (RT) for 30 min, and, after freezing in liquid nitrogen, were embedded in optimal cutting temperature media (Tissue-Tek; Sakura Finetek USA, Inc, Torrance, CA, United States). Sixty-micrometer frozen sections were then blocked in 2% bovine serum albumin containing 1% anti-CD16/CD32 Fc block (Bio X Cell, West Lebanon, NH, United States) for 90 min at RT and were stained with fluorophore-conjugated antibodies against CD3 and CD45R/B220 (both BioLegend, San Diego, CA, United States) for 60 min. After washing, samples were imaged via a FV10-ASW confocal microscope (Olympus).

### Flow Cytometry

Mandibular dLNs of CD11c-YFP mice were excised, and single cells were obtained by mechanically passing the samples through a 40 μm strainer (Thermo Fisher Scientific, Waltham, MA, United States). Single-cell suspensions were then blocked in FACS buffer containing 1% anti-CD16/CD32 Fc block (Bio X Cell) and were stained with a viability marker (LIVE/DEAD Fixable Blue Dead Cell Stain kit, Thermo Fisher Scientific) for 30 min at RT. Next, samples were stained with fluorophore-conjugated antibodies against CD45, CD11c, dendritic cell inhibitory receptor 2 (DCIR2), CD68, CD3, or respective isotype controls (all BioLegend, or BD Biosciences, San Jose, CA, United States) for 45 min at RT to yield fluorescence minus one stainings. After washing, samples underwent flow cytometric acquisition using a BD LSR II flow cytometer (BD Biosciences). Postacquisition data analysis was performed using FlowJo v9 software (FlowJo LLC, Ashland, OR, United States).

### Image Analysis

Images were exported from the multiphoton microscope to reconstruct 3D and 4D videos and to analyze the images with Imaris (Bitplane, Zurich, Switzerland). CD11c-YFP^+^ cells were counted, and densities calculated (cell number divided by the volume of the analyzed area of the dLN). Cell 3D instantaneous velocity, mean speed, displacement, and meandering index were obtained. Cell mean speed was defined as the mean speed of a cell over the entire measurement period (μm/min). Cell displacement was defined as the distance between first and last imaging point (μm). Meandering index, a measure for the directionality of cell migration, was defined as the ratio of the cell displacement and the cell's total path length (30). In all measurements of 3D instantaneous velocity, mean speed, displacement, and meandering index, tracks shorter than 2 min were excluded from analysis.

### Statistical Analysis

Results are presented as mean ± standard error of the mean (SEM). Three MP-IVM stacks/time series or confocal micrographs were analyzed from three different animals/condition and statistical significance determined by either *t*-test or one-way ANOVA with Tukey *post-hoc* (Prism Graphpad software, San Diego, CA, United States). Differences between groups were considered significant at *p* < 0.05.

## Results

### Components Necessary for Intravital Microscopy of the Mandibular dLNs

**Figure 1:**
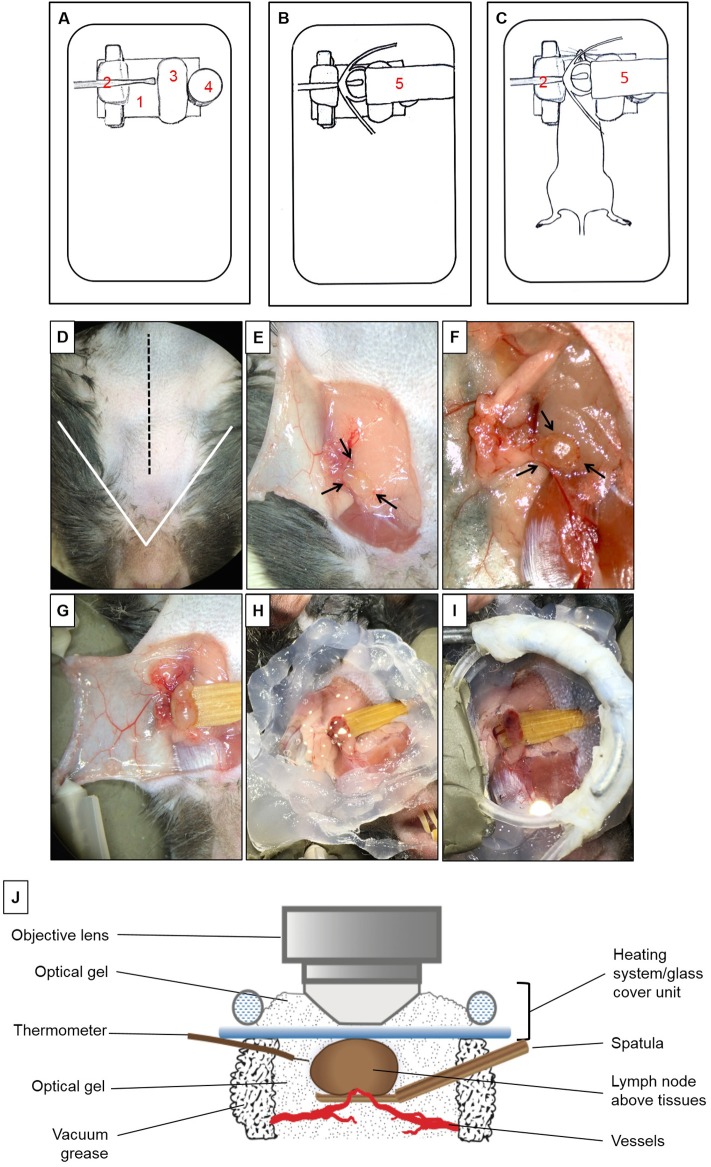
Multiphoton intravital microscopy setup. **(A–C)** Microscope stage setup for imaging of mandibular draining lymph nodes (dLNs) with multiphoton intravital microscopy (MP-IVM). **(A)** Partially assembled stage: (1) Metallic flat base to be used as support for the magnetic parts of the stage. (2) Spatula to keep the dLN slightly elevated above surrounding tissues with keeping nerves, blood, and lymphatic vessels intact. (3) Skin flap holder. (4) Magnetic pedestal for positioning of heating system. **(B)** Fully assembled stage: (5) Heating tubing system positioned on top of magnetic stand. This tube is connected to a peristaltic pump on one end, and the other end is immersed in a hot water bath to create a continuous source of heat around the dLN during intravital imaging. Below the tubing circle, a glass coverslip functions as the roof of a chamber in which the dLN is maintained moist and at the physiological temperature during imaging. **(C)** Positioning of anesthetized mouse on the completely assembled stage. **(D–I)** Microsurgical preparation of the mandibular dLN. **(D)** Ventral view of the surgical area (mouse's head toward the surgeon). The area above the white triangle represents *the regio intermandibularis*. The dotted black midline represents the area where an approximately 15 mm skin incision is made. **(E)** The skin flap is separated, exposing the ipsilateral mandibular dLNs (black arrows) and is secured with two 30G needles (shown in **D**) to keep the dLNs exposed at all times. **(F)** After microdissection, one of the dLNs is carefully liberated from surrounding tissues (arrows). **(G)** A wooden spatula is positioned underneath the dLN to slightly elevate it above surrounding tissues. Proper blood flow to the dLN can be surveyed with any necessary adjustments made at this step. **(H)** Vacuum grease is applied around the dLN to create the walls of a chamber and filled with optical gel. **(I)** Heating system/glass coverslip unit is positioned onto the grease surrounding the dLN, creating a sealed chamber containing the dLN and optical gel. **(J)** Schematic diagram of the dLN and MP-IVM imaging chamber highlighting the components of the chamber, positioning of the heating system/glass coverslip unit, and the thermometer. Through this unit, warm water recirculates at a constant rate to keep the chamber at desired 37 ± 1°C. This chamber is filled with optical gel to keep the moisture and adequate temperature around the dLN during imaging with MP-IVM. A thermometer is located inside the chamber to control the temperature during imaging.

To isolate the mandibular dLNs for MP-IVM, we utilized a custom-built microscope stage with a metallic base to provide support for the magnetic pieces of the stage (Figure 1A). A piece of modeling clay was used to secure the skin flap created during the microsurgical preparation and to support the mouse head laterally. A wooden spatula was used to keep dLNs slightly elevated above the surrounding tissues during intravital imaging, while nerves, blood, and lymphatic vessels were retained intact. A pedestal composed of three to four small magnets served as support for positioning a heating system unit on top of a dLN chosen for imaging, and the height of this stand was adjusted by adding or removing magnetic pieces according to the size of the mouse. A heating system/glass coverslip unit was used to regulate the dLN temperature during imaging and was made of polyethylene tubing (Intramedic, Sparks, MD, United States, Cat. No. 427420), looped to fit an 18-mm circle glass coverslip (VWR Cat. No. 48380046). One end of the tube was connected to a peristaltic pump (Masterflex L/S, Cole-Parmer, Vernon Hills, IL, United States), and the other end was immersed in a hot water bath. During image acquisition, hot water was continuously pumped through this system, maintaining the temperature of the surgical area at desired 37 ± 1°C. The fully assembled stage setup, without and with a mouse, are represented in Figures 1B,C.

### Microsurgical Preparation of Mandibular Draining Lymph Nodes for Multiphoton Intravital Microscopy

Mice were initially anesthetized with an intraperitoneal (i.p.) injection of ketamine HCl (100 mg/kg), xylazine (20 mg/kg), and acepromazine (3 mg/kg), resulting in up to 70 min of deep anesthesia. The proper plane of anesthesia was assessed every 30–60 min, and supplemental doses of ketamine (100 mg/kg) were given as required, alternating between ketamine/xylazine/acepromazine and ketamine. The fur on the ventral side of the neck was shaved, and 0.08 ml Bupivacaine 0.25% (Hospira, Inc., Lake Forest, IL, United States) was injected subcutaneously to minimize local pain/distress during the procedure. A 15-mm midline skin incision was made (Figure 1D). A skin flap was separated from the superficial cervical fascia to expose the ipsilateral mandibular dLNs (Figure 1E). This skin flap was fixed to the stage to keep the surgical area around the dLNs gently exposed (Figures 1F,G). Under a dissection microscope, the adipose tissue around one of the dLNs was removed without damaging the dLN vasculature (Figure 1F). To avoid artifacts due to minor movement of the dLN from breathing and heartbeats, a small wooden spatula was placed underneath the dLN to keep it slightly elevated and out of contact with surrounding tissues (Figure 1G and Supplementary Video 1). Special attention was paid to preserve blood circulation of the dLN when elevating the spatula. Vacuum grease (Dow Corning, Midland, MI, United States) was applied around the dLN and optical gel (Genteal gel, Novartis, Fort Worth, TX, United States) then applied on the dLN to maintain moisture during imaging (Figure 1H). The heating system/glass coverslip unit was then mounted on the grease, creating a sealed chamber over the dLN area (Figures 1I,J), and contact between the glass coverslip and the surface of the dLN. The heating system/glass coverslip unit allowed temperature regulation of the dLN with warm water circulated in a continuous motion with a peristaltic pump and water bath. The temperature of the surgical area inside the chamber, in close proximity to the dLN, was monitored throughout imaging with a dual input digital thermometer (Model HH12B, Omega Engineering, Norwalk, CT, United States; Figure 1J) and adjusted to 37 ± 1°C. During surgical preparation, the mouse body temperature was regulated with disposable hand warmers and foil.

### Multiphoton Intravital Microscopy of the Mouse Mandibular Draining Lymph Nodes

Once the dLN was properly prepared and a sealed chamber over the dLN was achieved, the animal was carefully placed under the microscope; the objective lens was positioned above the glass coverslip and dLN (Figure 1J). Optical gel was used to create an interphase between the objective lens and the glass coverslip. Through the eyepieces of the microscope, we could visualize the dLN under epifluorescence illumination before scanning, to ensure that the objective lens is in the correct position above the dLN. During image acquisition, the mouse body temperature was regulated with disposable hand warmers (HeatMax Inc., Dalton, GA, United States) and foil. The pulse rate, oxygen saturation, respiratory rate, and body temperature of the mouse were continuously monitored utilizing a MouseOx Plus monitor (Starr Life Sciences, Oakmont, PA, United States).

### Characterization of YFP^+^ Cells in the Mandibular Draining Lymph Nodes of CD11c-YFP Mice During Steady State

**Figure 2:**
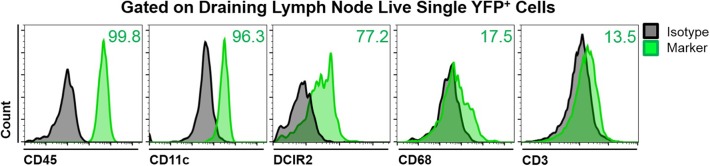
Characterization of YFP^+^ cells in mandibular draining lymph nodes of CD11c-YFP mice during steady state. Representative flow cytometric histograms showing coexpression of CD45 (pan-leukocyte marker), CD11c (cDC marker), DCIR2 (cDC marker), CD68 (macrophage marker), or CD3 (T cell marker) on live single YFP^+^ cells. Gray histograms represent fluorescence minus one isotype control staining and green histograms show coexpression of the indicated marker. Plots are representative of three independent experiments.

To assess the phenotype of YFP^+^ cells in the mandibular dLNs of transgenic CD11c-YFP mice, we analyzed coexpression of various immune cell markers on YFP^+^ cells during steady state, using fluorescence minus one staining followed by flow cytometry. We initially gated out debris, dead cells, and doublets, using forward and side scatters and a viability marker. As shown in Figure 2, YFP^+^ cells in the mandibular dLNs were mainly cDCs, as they coexpressed CD45 (pan-leukocyte marker), CD11c (cDC marker), and DRIC2 (cDC marker), with a small population (17.5%) coexpressing the macrophage marker CD68 and a minor fraction constituting T cells (CD3^+^; 13.5%). Thus, our flow cytometry evaluation indicated that YFP^+^ cells in the mandibular dLNs of CD11c-YFP mice were predominantly cDCs and, to lesser extent, macrophages or T cells.

### Multiphoton Intravital Imaging of Naïve CD11c-YFP^+^ Cells of the Mandibular Draining Lymph Nodes

**Figure 3:**
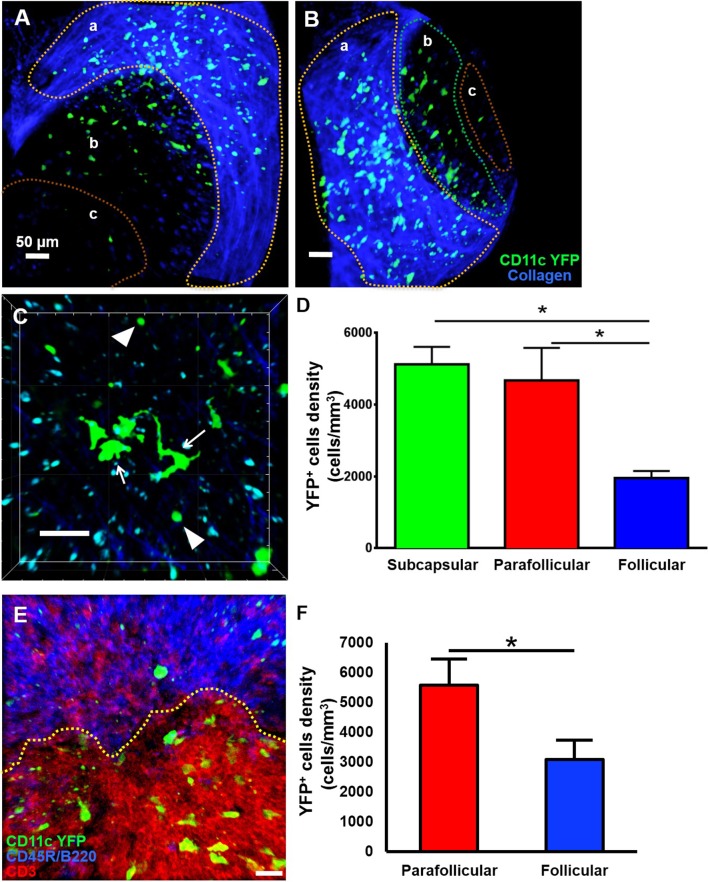
Anatomical distribution of CD11c-YFP^+^ cells in mandibular draining lymph nodes during steady state. **(A–D)** Representative multiphoton intravital microscopy images of a mandibular draining lymph node (dLN) during steady state. **(A)** Maximum projection of a mandibular dLN in which three areas can be identified: (a) subcapsular area delimitated by the second harmonic generation (blue; capsule's collagen fibers), (b) parafollicular cortex or T cell areas, and (c) follicular cortex or B cell areas. CD11c-YFP^+^ cells (green cells) are distributed predominantly in the subcapsular area, but also in the follicular and parafollicular areas. **(B)** Oblique view of the same dLN, in which the extent of the dLN's capsule (blue) can be appreciated. **(C)** Magnified parafollicular cortex of the dLN, showing different YFP^+^ cDC morphology and sizes. White arrows point to cells with dendritiform projections; arrow heads indicate round shaped, smaller cDCs. **(D)** Quantitative analysis of CD11c-YFP^+^ cell densities among the three areas of the dLNs during steady state (pooled from three independent experiments with a total of *n* = 3 mice). **(E,F)** Confocal microscopy of a mandibular dLN during steady state. **(E)** Representative confocal micrograph of a 60 μm section of a dLN of a CD11c-YFP mouse during steady state, showing distribution of CD11c-YFP^+^ cells (green) among parafollicular cortex or T cell area (CD3, red) and follicular cortex or B cell area (CD45R/B220, blue). **(F)** Quantification of CD11c-YFP^+^ cells in the parafollicular and follicular areas (pooled from three independent experiments with a total of *n* = 3 mice). Bars: Mean ± SEM, one-way ANOVA with Tukey *post-hoc* (for **D**) and *t*-test (for **F**), *p < 0.05, scale bars: 50 μm.

Little is known about the dynamics of cDCs in mandibular dLNs in an *in vivo* setting. Therefore, we first sought to investigate CD11c-YFP^+^ cells kinetics during steady state mandibular dLNs. The collagenous capsule of the dLN was visualized using second harmonics, allowing delimitation of the subcapsular, parafollicular (T cell areas), and follicular areas (B cell areas) of the dLN (Figures 3A,B and Supplementary Video 2). We found that steady state CD11c-YFP^+^ cells are predominantly oval in shape, with some showing an elongated dendritiform shape (Figure 3C and Supplementary Video 3). The CD11c-YFP^+^ cells were distributed mostly in the subcapsular area (Figures 3A,D; 5,123.66 ± 487.31 cells/mm^3^) but also in the parafollicular cortex or T cell areas (4,677.87 ± 903.54 cells/mm^3^), and sparsely in the follicular B cell areas (1,958.33 ± 193.72 cells/mm^3^). We next performed confocal microscopy of mandibular dLNs of CD11c-YFP mice, stained with CD3 (T cell marker) and CD45R/B220 (B cell marker) to unequivocally distinguish parafollicular and follicular areas (Figure 3E). As presented in Figure 3F, confocal microscopy confirmed our MP-IVM findings, showing higher densities of CD11c-YFP^+^ cells in the parafollicular area (5,572.97 ± 875.02 cells/mm^3^) compared to follicular area (3,089.19 ± 641.43 cells/mm^3^; *p* = 0.02). Longitudinal analysis revealed static CD11c-YFP^+^ cells, exhibiting neither cell displacement nor dendrite extension, and motile CD11c-YFP^+^ cells with sampling motion (Supplementary Videos 3, 4). The average CD11c-YFP^+^ cells' mean speed during steady state was 3.58 ± 0.09 μm/min, where cells with smallest dimensions exhibited the most motility (Supplementary Video 4).

### Corneal Allotransplantation Resulted in a Significant Increase in Host-Derived CD11c-YFP^+^ Cells Kinetics in the Mandibular Draining Lymph Nodes

**Figure 4:**
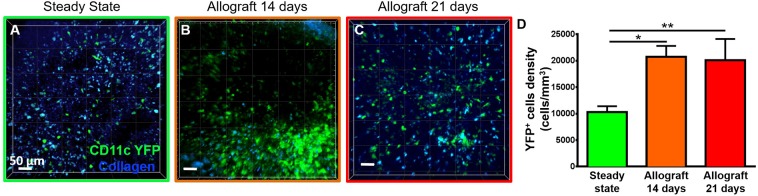
Density of CD11c-YFP^+^ cells in mandibular draining lymph nodes. **(A–C)** Representative multiphoton intravital microscopy images of mandibular draining lymph nodes (dLNs) during **(A)** steady state and **(B)** 14 and **(C)** 21 days after corneal allotransplantation reveal an increase in the number of host-derived CD11c-YFP^+^ cells (green cells). **(D)** Quantitative analysis of host CD11c-YFP^+^ cells density in dLNs (pooled from three independent experiments with a total of *n* = 3 mice/group). Bars: Mean ± SEM, one-way ANOVA with Tukey *post-hoc*, *p < 0.05, **p < 0.01, scale bars: 50 μm.

**Figure 5:**
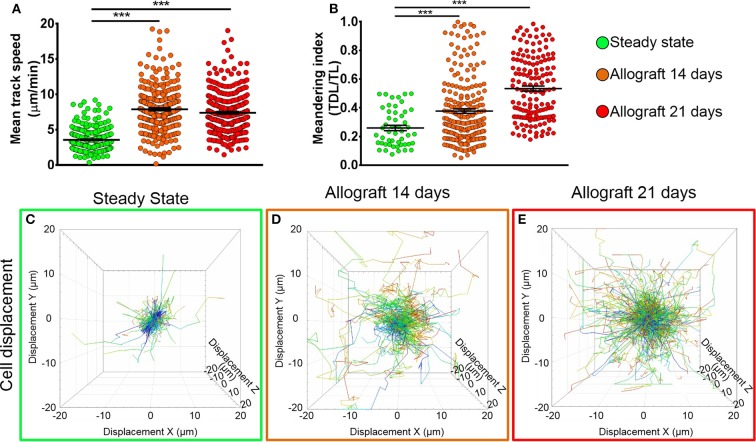
Kinetics of CD11c-YFP^+^ cells in mandibular draining lymph nodes. **(A,B)** Quantitative analysis of **(A)** mean speed and **(B)** meandering indices (track displacement length divided by track length) of host CD11c-YFP^+^ cells during steady state, 14, and 21 days after corneal allotransplantation (pooled from three independent experiments with a total of *n* = 3 mice/group). **(C–E)** Representative cell displacement lengths normalized to a starting point. Bars: Mean ± SEM, one-way ANOVA with Tukey *post-hoc*, ***p < 0.001.

To study the behavior of activated host cDCs in mandibular dLNs, we analyzed kinetics of CD11c-YFP^+^ cells at 14 and 21 days after corneal allotransplantation (allogeneic), as previously described (35). CD11c-YFP^+^ cell density in the mandibular dLNs increased significantly 14 days after corneal allotransplantation, compared to steady state (20,745.67 ± 2,050.87 vs. 10,307.56 ± 1,103.13 cells/mm^3^, *p* = 0.002; Figure 4). Cells agglomerated into the T cell areas (parafollicular cortex) and the majority acquired an oval shape; a few still conserved the dendritiform shape, with dendrites' probing movements more than real cell body movements (Supplementary Videos 3, 5). Analysis of cell motility revealed that host-derived CD11c-YFP^+^ cells exhibited increased mean speed and displacement (Figure 5). CD11c-YFP^+^ cells acquired higher speeds at day 14 following allotransplantation, compared to steady state (7.90 ± 0.19 vs. 3.58 ± 0.09 μm/min, respectively, *p* < 0.001; Figures 5A,C–E and Supplementary Video 6). Increased cell displacement and mean speed changes were maintained at 21 days after corneal allotransplantation (7.40 ± 0.17 μm/min; Supplementary Video 7).

We next aimed to investigate the meandering index of CD11c-YFP^+^ cells, defined as the ratio of the net displacement of a cell (from start to finish points in a straight line) and the total path length that the cell moved from the beginning to end (30). Interestingly, we found meandering indices of CD11c-YFP^+^ cells increased with time, 0.37 ± 0.01 at 14 and 0.53 ± 0.01 at 21 days postallotransplantation compared to steady state (0.26 ± 0.02, *p* = 0.001; Figures 5B–E). This indicates that CD11c-YFP^+^ cells exhibited a more directional migration pattern following transplantation compared to the random movement in the naïve mandibular dLNs.

### T Cells Demonstrate Distinct Kinetics Compared to CD11c-YFP^+^ Cells in the Mandibular Draining Lymph Nodes

**Figure 6:**
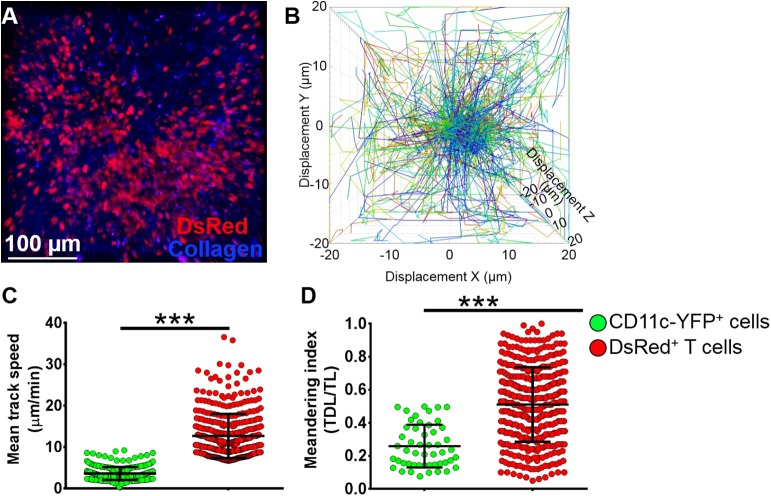
Kinetics of T cells in mandibular draining lymph nodes. **(A)** Representative multiphoton intravital microscopy image of a mandibular draining lymph nodes (dLNs) of a transgenic T-Red mouse during the steady state showing DsRed^+^ T cells (red) and collagen (blue). **(B)** Representative cell displacement lengths normalized to a starting point. **(C,D)** Comparison of cell mean speed **(C)** and meandering indices **(D)** between CD11c-YFP^+^ and DsRed^+^ T cells in the dLNs during steady state (pooled from three independent experiments with a total of *n* = 3 mice/group). Bars: Mean ± SEM, *t*-test, ***p < 0.001, scale bar: 100 μm.

To assess if our model would allow studying other immune cells as well, we next evaluated the kinetics of T cells in the mandibular dLNs during steady state (Supplementary Video 8). As shown in Figure 6A, we observed DsRed^+^ T cells (in red) in clusters in the dLN with collagen visualized by second harmonic generation (in blue). Figure 6B illustrates T cell displacement in the dLN during steady state. We observed brisk T cell meanderings in the mandibular dLNs with 3D instantaneous velocity of 6.08 ± 0.09 μm/min. We observed that, compared with CD11c-YFP^+^ cells (3.58 ± 0.09 μm/min), DsRed^+^ T cells displayed higher mean speed (12.67 ± 0.27 μm/min, *p* < 0.001; Figure 6C) and directionality, as well as a higher meandering index of DsRed^+^ T cells (0.51 ± 0.01) compared with CD11c-YFP^+^ cells (0.26 ± 0.02, *p* < 0.001; Figure 6D). Thus, our findings suggest that our model can be utilized to study kinetics of various immune cells in the mandibular dLNs.

## Discussion

Corneal immune responses have previously been widely studied in *ex vivo* settings. These studies have provided valuable insights about immune responses to foreign antigens. However, to understand the complexity of the corneal immune arc and the underlying cellular mechanisms involved in antigen presentation process to prime T cells and evoke an immune response, it is essential to study it in an *in vivo* context. During *in vivo* assessment, physiological parameters such as proper tissue oxygenation and temperature must be preserved, allowing cells to behave similarly to a physiological state. Herein, we present, to our knowledge, the first model for *in vivo* imaging of the corneal dLNs utilizing MP-IVM.

MP-IVM has been proven to be a reliable method to visualize and analyze innate and adaptive immune responses at a single-cell level. The more accessible inguinal and popliteal LNs have been widely studied via MP-IVM. The first report of *in vivo* imaging of the inguinal dLNs was in 2003, where Miller et al. described and quantified the migratory behavior of adoptively transferred fluorescently labeled T cells in mice (22). One year later, Mempel et al. described a mouse model to image popliteal dLNs utilizing MP-IVM (25). These reports reveal the feasibility and reproducibility of MP-IVM in imaging superficial dLNs. Contrary to these dLNs, *in vivo* imaging of mandibular dLNs is challenging due to its anatomical proximity to lungs and heart. Thus, respiratory movement and heartbeats are powerful mechanical artifacts that hinder a proper stabilization of the dLN for appropriate image acquisition at a microscopic level over time, since a few micrometers tissue movement can turn into uninterpretable 3D images (36). We have overcome this problem by stabilizing the mandibular dLN in two steps: careful microdissection of the tissue around the dLN and slightly elevating the dLN from/above the surrounding structures using a spatula. This in turn means imaging is not affected by heartbeats or breathing artifacts. During this step, it is crucial to ensure that the blood flow to the dLN is not interrupted by either severing or stretching of the feeding vessels while elevating the dLN. This is to ensure proper tissue oxygenation and maintaining proper physiological temperature (37 ± 1°C). Temperature is a critical aspect in analyzing cell dynamics: if proper temperature is not achieved or if it is not constant during the entire image acquisition, interstitial cell motility will be affected (23, 30), with no dendrite movement or “probing;” hence, dynamic analysis will not be reliable. We achieved a proper temperature, ensuring that a sealed chamber around the dLN was made with vacuum grease and was filled with optical gel. The function of this gel is to keep the dLN moist and at a physiological temperature. The ceiling of this chamber was formed by a glass coverslip and a heating tubing system through which hot water circulated at a constant rate. With this heating system, we tried attempted to mimic the physiological conditions of proper temperature for an adequate interstitial cell movement.

Through utilizing a custom-made stage, appropriate microdissection, a sealed chamber, and constant physiological temperature regulation, we have been able to perform longitudinal MP-IVM of the mandibular dLNs. We demonstrate that the mean speed and movement directionality of host-derived CD11c-YFP^+^ cells increase significantly after corneal allotransplantation as compared with steady state. Furthermore, after 14 days, CD11c-YFP^+^ cells tend to agglomerate in the parafollicular cortex of dLNs. This is in line with a previous report from Lindquist et al. who described the spatiotemporal characteristics of dendritic cell networks in normal inguinal dLNs of live mice (20). In these studies, the authors explained how steady state cDCs are entangled in an extensive cell network while they are probing T cells, but that after activation, these cells become more motile and distribute into the sessile network to be readily accessible to migrating T cells. Similarly, with our model of *in vivo* imaging of corneal dLNs, we are able to visualize the distribution of CD11c-YFP^+^ cells in all three regions of the mandibular dLNs. During steady state, CD11c-YFP^+^ cells are either static with some dendrite probing or exhibit a low mean speed with random movement throughout the dLNs parenchyma.

Previous studies have shown that corneal trauma results in secretion of proinflammatory cytokines, leading to activation of APCs, enhanced expression of maturation markers, including major histocompatibility complex II and costimulatory molecules, CD80 and CD86, subsequently resulting in their mobilization to dLNs (8, 37, 38). In the current study, we assessed the density and kinetics of CD11c-YFP^+^ cells in the mandibular dLNs following corneal transplantation. We observed that following corneal allotransplantation, density and motility of host CD11c-YFP^+^ cells increase significantly, with cell movement becoming more directional. The change in directionality may be indicative of CD11c-YFP^+^ cells movements toward T cells in the parafollicular areas of the dLNs to prime them and elicit T cell responses. Although we demonstrate alterations in the density and motility of CD11c-YFP^+^ cells, as a subpopulation of APCs in the mandibular dLNs following corneal transplantation, our study is limited in revealing potential association between alterations in cell kinetics and functions of these cells. Thus, further studies are necessary to examine the correlation between kinetics and functionality of APCs during steady state and following transplantation.

In the current study, T cells in the mandibular dLNs showed a mean track speed of 12.67 μm/min and 3D instantaneous velocity of 6.08 μm/min. In line with our findings, Miller et al. observed that average T cell speed in inguinal dLNs ranges between of 10.2 and 11.5 μm/min in live mice (22), and Mempel et al. reported mean 3D velocity of 8.4 μm/min for T cells in popliteal dLNs (23, 25). Furthermore, consistent with our findings, a study on kinetics of CD11c-YFP^+^ cells in the inguinal dLNs suggest that cDCs move more slowly compared to T cells with their median speed ranging between 1 and 4 μm/min depending on the dLN area (20). Indeed, MP-IVM has been used to study cell-to-cell interactions, a crucial step in understanding immune responses. In 2004, Mempel et al. could distinguish three stages of T cell activation mediated by DCs in popliteal dLNs over time (transient encounters, followed by stable contacts, and finally high motility and proliferation of T cells) (25). MP-IVM can be also used to study immune cells in peripheral tissues. For instance, in 2011, Celli et al. using a murine ear skin graft model and MP-IVM, showed that activation of graft-reactive CD8^+^ T cells occurs in the dLNs, and this T cell cross-priming is mediated by host's APCs that had previously infiltrated the graft (28). Furthermore, the study provided insight into molecular mechanisms involved in skin graft rejection. In a more recent report, Kitano et al. characterized the dynamic interaction of different cDC subsets in the skin dLNs, providing *in vivo* evidence of the differential role of migratory and resident cDCs in cytotoxic T cell generation within the dLNs (21). With our model of *in vivo* imaging of the corneal dLNs and utilizing different combinations of transgenic mice (for example, mice expressing fluorescent proteins subpopulations of T cells), one would be able to study the process of antigen presentation in inflamed and pathological states of the cornea, such as after corneal transplantation, and may be able to characterize the dynamics and molecular mechanisms involved in corneal graft rejection to provide insights that may lead to the development of immunomodulatory therapies to prevent immunological transplant rejections.

## Data Availability Statement

The datasets generated for this study are available on request to the corresponding author.

## Ethics Statement

The animal study was reviewed and approved by Schepens Eye Research Institute Animal Care and Use Committee and Tufts Medical Center Animal Care and Use Committee.

## Author Contributions

ML, TY, and PH designed the research. ML, TY, AJ, VS, and GO performed the research. ML, YS-R, TY, AJ, GO, DH, and PH analyzed the data. ML, YS-R, AJ, VS, DH, and PH wrote the paper.

### Conflict of Interest

The authors declare that the research was conducted in the absence of any commercial or financial relationships that could be construed as a potential conflict of interest.

## Supplementary Material

Supplementary Video 1 After careful microdissection of tissue surrounding the dLN, and without damaging feeding vessels, the dLN is positioned on a wooden spatula, and slightly elevated above surrounding structures. In this video, the dLN has been slightly repositioned to show intact vasculature, uninterrupted blood flow, and heart pounding or breathing movements that would otherwise impede imaging are not observed.

Supplementary Video 2 Mandibular dLN of a CD11c-YFP mouse during steady state. Z-stack of optical sections rendered into 3D data set. The dLN capsule's collagen fibers can be observed through the second harmonic generation (blue). Located underneath the collagen capsule are the CD11c-YFP^+^ cells (green) in the subcapsular area (a). Toward the center, is located the parafollicular area or T cell zone (b) and the follicular area or B cell zone (c), where CD11c-YFP^+^ cells are still present, but sparsely. Scale bar: 50 μm.

Supplementary Video 3 Zoomed parafollicular cortex of a mandibular dLN of a CD11c-YFP mouse during steady state. Z-stack of optical sections rendered into 3D data set. Different cell morphologies and sizes of CD11c-YFP^+^ cells can be appreciated (green). Scale bar: 50 μm.

Supplementary Video 4 Time-lapse video of the parafollicular area of a mandibular dLN of a CD11c-YFP mouse during steady state. CD11c-YFP^+^ cells (green) are distributed throughout the dLN parenchyma. The majority of them show static cell bodies, with dendrite movement or “probing.” The smaller cells are more motile, showing random movements with larger displacements. Scale bar: 50 μm.

Supplementary Video 5 Time-lapse video of the parafollicular area of a mandibular dLN of a CD11c-YFP mouse during steady state. Zoomed to appreciate CD11c-YFP^+^ cells dendrites' “probing” and movement of the smaller cells through the dLN parenchyma. Compared with the larger ones, the smaller cells are more motile, interacting and making close contacts with each other. Scale bar: 50 μm.

Supplementary Video 6 Time-lapse video of the parafollicular area of an ipsilateral mandibular dLN 14 days after corneal allotransplantation to a CD11c-YFP mouse. CD11c-YFP^+^ cells (green) are distributed throughout the dLN parenchyma, some of them clustered in the inferior right corner. Scale bar: 50 μm.

Supplementary Video 7 Time-lapse video of the parafollicular area of an ipsilateral mandibular dLN 21 days after corneal allotransplantation to a CD11c-YFP mouse. CD11c-YFP^+^ cells (green) are distributed throughout the dLN parenchyma, most of them have lost their dendritiform shape, and have become oval, representing smaller and more motile. Scale bar: 50 μm.

Supplementary Video 8 Time-lapse video of the parafollicular area of a mandibular dLN of a transgenic T-Red mouse with DsRed^+^ T cells during steady state. T cells (red) show random rapid movements with large displacements. Scale bar: 50 μm.
